# The legacy and evolvability of Pere Alberch’s ideas

**DOI:** 10.1098/rsfs.2024.0011

**Published:** 2024-10-25

**Authors:** Laura Nuño de la Rosa, Gerd B. Müller

**Affiliations:** ^1^ Department of Logic and Theoretical Philosophy, Complutense University of Madrid, Madrid, Spain; ^2^ Theoretical Biology Unit, University of Vienna, Wien, Austria; ^3^ Konrad Lorenz Institute of Evolution and Cognition Research, Klosterneuburg, Austria

**Keywords:** Pere Alberch, evo-devo, developmental constraints, novelty, evolvability

## Abstract

Pere Alberch played a pivotal role in shaping the field of evolutionary developmental biology during the 1980s and 1990s. Whereas initially his contributions were sidelined by the empirical advancements of the molecular revolution in developmental and evolutionary biology, his theoretical insights have left a lasting impact on the discipline. This article provides a comprehensive review of the legacy and evolvability of Alberch’s ideas in contemporary evo-devo, which included the study of morphogenesis as the proper level of developmental causation, the interplay between developmental constraints and natural selection, the epistemic role of teratologies, the origin of evolutionary novelties and the concept of evolvability.

## 1. Introduction

The emergence of evolutionary developmental biology as a discipline is often attributed to the discovery of shared genes, Hox genes in particular, and of homologies in gene regulatory pathways across vastly distant phyla. However, historical reconstructions of science tend to be influenced by contemporary scientific paradigms, favouring narratives that conform with the prevailing approaches at the time of examination. The current dominance of the molecular approach in the study of the relationship between evolution and development is a case in point. According to this paradigm, evo-devo is essentially comparative developmental genetics, representing a convergence of phylogenetics and developmental genetics.

In contrast to this widespread view, many authors emphasize that evo-devo is a very heterogeneous field in which different research traditions coexist. A number of studies demonstrate that evo-devo did not merely evolve from descriptive embryology into comparative developmental genetics (e.g. [1,2]). Rather, the field arose from the junction of several research strategies and traditions, including both molecular and morphological ones. This multidisciplinary ethos was integral to the 1981 Dahlem Conference organized by John Bonner, a seminal gathering that recognized the fusion of ideas from diverse domains as essential to exploring the nexus between development and evolution. The resulting edited volume [3] would serve as a linchpin for evo-devo’s advancement over the ensuing decades (see [4]).

The young Catalan evolutionary biologist Pere Alberch (1954–1998) was one of the participants at that meeting. The work that he would develop over the subsequent two decades is an outstanding example of disciplinary cross-fertilization among research traditions outside genetics. For this reason, he cannot be considered merely a precursor of modern evo-devo, but especially as someone who advanced his own morphogenetic approach alongside the rapid rise of molecular evo-devo, remaining well aware of its methodological innovations and new phylogenetic findings. He chose a different strategy—an integrative approach that combined theoretical and functional morphology, natural history, experimental embryology and mathematical modelling—to answer questions neglected by the neo-Darwinist framework. These included long debated topics in the morphological tradition such as homology, homoplasy and novelty but also new ones such as those related to the evolvability of developmental systems. It is precisely this convergence of traditions that renders Alberch’s thinking particularly innovative since it unveiled previously uncharted intellectual spaces.

**Figure 1.**
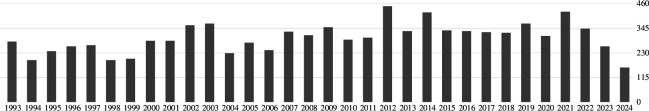
Citations of Pere Alberch’s works per year. Source: Google Scholar.

Pere Alberch’s life and intellectual trajectory have been thoroughly examined in prior works [5–8], and our intention here is not to replicate these efforts but to build upon the existing scholarship in order to analyse the lasting impact of Alberch’s ideas on the evolution of evo-devo. Whereas some authors’ contributions exhibit bursts of popularity during specific periods in the history of a discipline, in other instances citations do not spike dramatically but maintain a steady level over time. This pattern is observable in the case of Alberch, whose work has captured the interest of evolutionary biologists across various disciplinary domains up to the present day (figure 1). As will become apparent throughout this article, the enduring significance of his work is based not so much on his empirical investigations but predominantly on his theoretical contributions. It is in this sense that we claim that Alberch’s ideas not only evolved but were evolvable, insofar as they anticipated many of the debates that would shape the development of evo-devo in the subsequent decades. One key discussion revolves around the conceptualization of development itself and its causal influence on evolution as derived from this reasoning. In the next section, we will contrast Alberch’s views on evolutionary causation with the mainstream molecular perspective in evo-devo. Subsequently, we will discuss his evolving opinion on developmental constraints through his investigations of morphogenetic systems and the study of teratologies. In the last sections, we will analyse the role of constraints in current evolutionary debates, Alberch’s intellectual shift towards evolvability and the further evolution of this concept.

## 2. From genes to developmental systems: the morphogenetic approach

**Figure 2.**
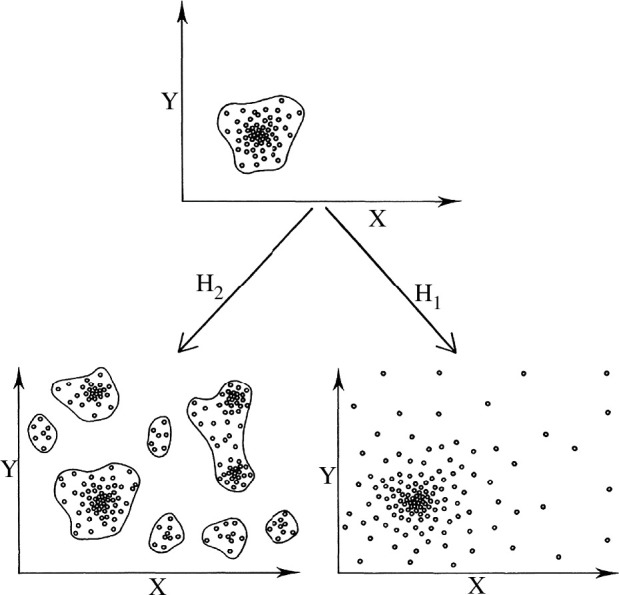
Schematic illustration of the effects of developmental constraints on the distribution of morphological variation, reproduced from Alberch [13], used with permission from Springer. This figure depicts a hypothetical experiment on the evolution of a phenotype, as described by Alberch [13, pp. 315–319]. The upper panel shows the initial morphological states of a population. If the action of selection is reduced to a minimum through generations of random mating (eliminating competitive interactions, etc.), two potential patterns emerge. Hypothesis 1 (H_1_) suggests that random variation fills most of the possible morphology space, while hypothesis 2 (H_2_) proposes that development channels genetic variability into a discrete subset of morphological states.

Alberch’s work supports the historiographical argument that morphology was central to the emergence of evo-devo as a discipline [9,10]. Influenced by David Raup’s theoretical morphology [11] and Gould & Lewontin’s critique of adaptationism [12], Alberch observed that phenotypic variants are not uniformly distributed in a species’ morphospace, as they occupy only a subset of the conceivable forms in the absence of selection (see figure 2). In contrast to the conventional perspective in population genetics, which assumes that phenotypic variations distribute isometrically as a result of random mutations later fixed by natural selection, the structure of morphological variation suggests that it is not only the processes retaining that variation but also the mechanisms generating it that need to be taken into account [13,14].

Alberch’s contributions played a crucial role in reintegrating morphology into evolutionary theory [5,15]. Taking morphology seriously was essential for building the argument that an understanding of evolution required an understanding of development. However, the meaning of development was not unequivocal at that time, and this is precisely what continues to be at stake in the different current ways of understanding and practising evo-devo. Early in his career, Alberch explored heterochrony (i.e. evolutionary changes in the timing or rate of development) as a key mechanism in evolutionary change. Stephen J. Gould revived the interest in heterochrony with his seminal 1977 book *Ontogeny and phylogeny* [16], and Alberch contributed to this resurgence (see [17,18]) with his landmark paper ‘Size and shape in ontogeny and phylogeny’ [19]. Co-authored with Gould himself, George Oster and David Wake, it was his first international publication, and it became one of the most referenced works in the history of evo-devo. The article elaborated on Gould’s clock model, redefining heterochrony as emerging from changes in developmental processes rather than from comparisons of the phenotypic outcomes. Since then, the recognition of heterochronic patterns and processes has illuminated our understanding of animal evolution, with salamanders remaining the group that has arguably contributed the most in this regard (see [18]).

Despite this quantitative and process-based reformulation of heterochrony, Alberch soon became dissatisfied with the idea that evolution proceeded through the temporal modification of development. According to him, the approach retained a ‘static’ and ‘descriptive’ attitude, echoing the traditional recapitulationist view of comparative morphology, which saw development as a sequence of discrete stages preserved in evolution. He argued that if we wanted to understand the changes between related morphologies, the traditional focus on embryological stages should be replaced by a dynamical and causal approach that investigated *developmental rules* rather than discrete points in a continuum [20–22].

In line with the experimental embryology tradition and in contrast to the conventional view of evolution in which the origin of variation is taken for granted, Alberch advocated ‘mechanistic’ investigations to elucidate the processes by which forms arise in development. However, instead of focusing on genes, Alberch chose a *morphogenetic* approach that centred on global properties of developmental networks [23]. According to this innovative framework, patterns of variation emerge as an inherent consequence of developmental properties, primarily driven by epigenetic (dynamical) interactions occurring at the cellular level [20,24]. Organismal form emerges from internal ‘construction rules’ of developmental systems, and it is in the context of these systems that mutations and environmental changes influence phenotypic outcomes. As we will see in the next section, the evolutionary perspective permits understanding the emergence, maintenance and ‘generative capabilities’ of the construction processes that underlie altered morphological outcomes [13].

The morphogenetic approach contrasts sharply with the developmental genetics position on developmental causality and the associated methodology used to unveil it [25]. Developmental genetics typically perceives development as a sequence of gene expressions revealed through an interventionist methodology wherein induced genetic alterations yield discrete phenotypic changes. This procedure traces a chain of events from perturbation to consequence, attributing causal significance to the altered gene in the developmental process. From an organismic perspective, this approach has significant limitations: while it identifies certain causal elements, it fails to elucidate the complex suites of interactions that determine the final phenotypic outcomes. Alberch’s morphogenetic perspective embraced a reciprocal and interdependence-based understanding of developmental causality that explicitly rejected its reduction to gene expression. Instead, genes were taken to endow cells with specific properties such as adhesivity, or migratory or proliferative capacity, which then self-organize guided by physico-chemical principles to generate structures and organs [26]. Morphogenetic motifs emerging from interactions at molecular, cellular and tissue levels were not seen as genetically preprogrammed but as arising from the dynamical nature of developmental systems [21]. Alberch’s work followed in the wake of D’Arcy Thompson’s allometric conception of organismal form and of the ideas of chemical morphogenesis developed by Turing [27] and further elaborated by Gierer & Meinhardt [28] and Palmquist *et al*. [29]. But he extended these concepts by defining rules of cellular construction and applying them to evolutionary contexts. From a methodological standpoint, Alberch’s experimental approach targets interventions at the morphogenetic level to uncover the cellular behaviours of developmental systems, together with an examination of their formal properties using models derived from dynamical systems theory.

Today, the bulk of evo-devo studies fall under the umbrella of molecular evo-devo, reflecting the prevalence of reductionist strategies in developmental biology. Since the 1990s, the type and number of key regulatory genes and networks invoked in explanations of development, and consequently of organismal evolution, have increased dramatically. While transcription factors were once central, new molecular factors such as epigenetic modifications of DNA and the ubiquitous microRNAs have been added to the ‘developmental genetic toolkit’ [30]. Because of the molecular nature of these advancements, the evolutionary framework also remains largely focused on molecular causality, with few studies embracing a morphogenetic approach akin to that of Alberch. While the prevalence of molecular biology has indeed eclipsed his contributions to evo-devo, recent years have witnessed a revival of morphology in biology and biophysics, returning Alberch’s work to prominence [6]. In particular, there has been a resurgence in biophysical investigations of morphogenesis over the last few decades [31], with Alberch being acknowledged as a significant ideator of this field (Palmquist *et al*. [29]).

Recent methodological advances, such as the emergence of new imaging and computational techniques, have facilitated this resurgence of interest [32]. Alberch belonged to the predigital age when studies of development were still derived from microscopical observations that relied on visual inspection and the individual analysis of photographs. Since the mid-1990s, digitization and the exponential increase in image resolution have dramatically transformed the reconstruction of developmental processes at the molecular, cellular and tissue levels [33]. Whereas in developmental genetics images are mostly considered mere illustrations of the effects of genes in development, in biophysical explanations of morphogenesis the new imaging methodologies assume a crucial role, insofar as the characterization of developmental processes with high spatial and temporal resolution (e.g. cell migratory tracks and cell division patterns) facilitates the formulation of biophysical models of cell behaviour and mechanics [34].

The conference ‘Pere Alberch, 25 years on: genes, cells, and embryos in development and dvolution’, held in Barcelona in November 2023, was testimony to the evolution of evo-devo and of Alberch’s pervasive influence on the field. It also sparked the present special issue. The conference brought together pioneers of the molecular approach to evo-devo, such as Peter Holland, Denis Duboule or Cliff Tabin, with organism-focused scientists, such as Frietson Galis and Neil Shubin, as well as representatives of a new generation of evo-devo researchers endorsing integrative, multiscale approaches. The latter proves particularly relevant to understanding genotype–phenotype (GP) relationships. Patrick Lemaire, for instance, presented the case of ascidian embryos that develop along similar morphogenetic pathways despite possessing very different genomes [35]. Other species, such as cichlids, show the inverse condition, exhibiting substantial morphological diversity in the face of practically identical genomes [36]. Both lines of evidence converge in outlining that the GP map is nonlinear and that the reasons for this nonlinearity are to be found in developmental interactions operating at scales beyond the molecular level. Jim Sharpe’s exploration of the developmental evolution of vertebrate limbs underscored the significance of multiscale computer modelling in comprehending the intricate relationship between molecular processes and tissue-level dynamics [37]. Vertebrate limb development was already a preferred model system used by Pere Alberch for gaining insight into how organogenesis across different scales governs morphological evolution. But whereas his approach was considered bizarre at the time, today a combination of experimental embryology, advanced imaging technologies and mathematical modelling has become the favoured strategy in the study of the mechanisms that underlie the emergence and evolution of phenotypes.

## 3. Developmental constraints and the logic of monsters

When Gould & Lewontin [12] introduced the notion of ‘developmental constraint’, their aim was to criticize the view of evolutionary adaptation as the process of optimization that is responsible for all phenotypic characters observed. In their approach, constraint did not refer to how evolving levels of organization integrate into a restrictingly complex system, but it emphasized the limitation of the powers of natural selection. This interpretation was modified by Alberch and others, who saw developmental constraints as limitations on the production of variant phenotypes, i.e. a limitation of the generative, not of the selective process. Since then, the concept of constraints on evolution has been caught in a struggle between these two difficult-to-conciliate senses of limitation, that of restrictions on natural selection and that of restricted phenotypic variation [7]. As we will discuss in the following section, it remains an open question until today whether these two conceptualizations of constraint are mutually exclusive, or whether they represent two perspectives of the same phenomenon.

According to Alberch [13,23], constraints reflect the intrinsic tendencies of developmental systems to produce certain forms rather than others. As a consequence of the view of developmental causation discussed above, these constraints are not reducible to genetic restrictions, but derive from the properties of multiple levels of developmental organization, such as entrenched gene regulatory cascades, the autonomous capacities of cells to self-organize, the inductive interactions among embryonic regions, the topology and geometry of cell populations or the physical properties of cells and tissues, to name but a few. Any such system of multiple interdependencies among its constituent elements will exhibit particular responsive dynamics upon perturbation. Regardless of whether disturbances are environmental, selectional, mutational or experimental, the morphogenetic reaction norms will determine a limited number of phenotypic outcomes, or, as Alberch would have put it, ‘the morphospace will be discrete’. It also means that morphogenetic systems can be explored experimentally to yield the inherent propensities that are of evolutionary relevance.

**Figure 3.**
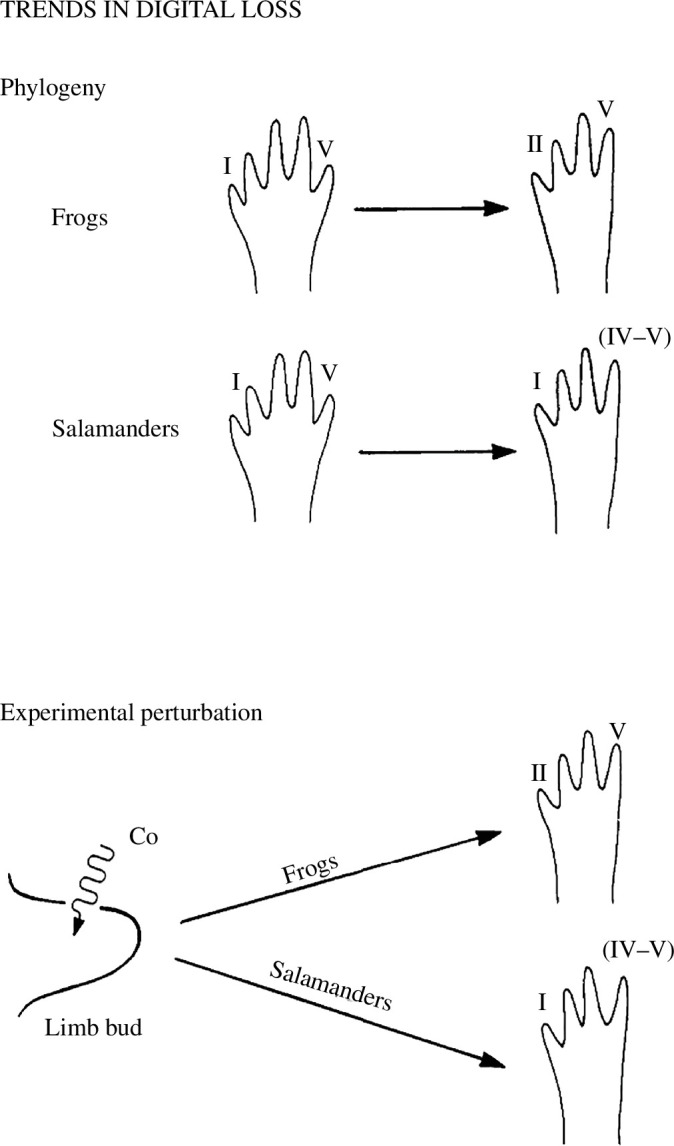
Summary of trends in digital loss, reproduced from Alberch & Gale [38], used with permission from Oxford University Press. From a phylogenetic perspective, frogs lose their first toe, whereas salamanders lose their toes on the postaxial side (digits IV and V). This pattern can be replicated experimentally by reducing the cell count in the limb bud through the application of a mitotic inhibitor, such as colchicine (Co).

Alberch’s work on constraints in amphibian digital reduction has become the most cited example of an evo-devo hypothesis. In two papers, Alberch & Gale [24,38] proposed that the different trends of digital reduction observed in frogs and salamanders are due to different labilities of the digit-forming systems in the two lineages (see figure 3). They characterized the phylogenetic trends using descriptive comparative data and explored the underlying developmental processes by submitting embryonic limb primordia of both lineages to treatments of mitotic inhibition. The resulting reductions of cell number in the growing limbs led to the preferential loss of preaxial digits (digit I) in frogs and of postaxial digits (digits V and IV) in salamanders, mimicking the phylogenetic sequences of digit loss recurring in multiple species of salamanders and frogs, respectively. The experimental loss of individual phalanges showed a more differentiated picture but also largely matched the tendencies observed in the phylogenetic losses in both lineages.

From their experiments, Alberch and Gale deduced several morphogenetic rules. First, regardless of which pattern-forming mode of limb development is assumed, there exists a linkage between the size of the involved embryonic fields and the resulting patterns. The relevant quantitative differences are based on the number of cells available within the limb primordia. Second, phylogenetically, anurans and urodeles have evolved different sequences of digit formation, but reductions in the number of cells that contribute to the digital anlagen are answered in the same way: primordia forming last will disappear first. Hence, a lineage-specific response (digit loss), resulting from the same perturbation (limb size reduction), may be initiated by different kinds of selective processes, such as overall miniaturization or local changes in cell proliferation rates. Finally, morphological change is not necessarily linked to specific genetic change. Mutations may affect many different morphogenetic parameters, such as cell proliferation, adhesivity or migration, but via their common effects on cell number, they can all produce the same morphogenetic outcome.

Alberch’s ideas on digit reduction did not go uncontested. Galis *et al*. [39] claim that evolutionary digit reduction in vertebrates happens as a consequence of the active destruction of a digit rudiment that had already formed and that the maintenance of the basic pentadactyl pattern is a consequence of pleiotropic genetic constraints. The explanandum in Alberch’s study, however, was why one lineage of amphibians preferentially loses preaxial digits, whereas another lineage preferentially loses postaxial digits. To relegate this question to potential pleiotropic interdependencies (or the lack thereof) is a prime example of a clash of the notion of evolutionary constraints (natural selection cannot easily alter digit number) with that of a developmental constraint (the sequence of phylogenetic digit reduction is determined by developmental propensities). The latter applies even in cases in which digit reduction occurs not by the failure of a digit rudiment to form but by its later suppression. Although apoptosis or necrosis have not been shown to take place in already formed digit anlagen, the discontinuation of the growth of a digit rudiment is one of the known mechanisms of evolutionary digit reduction. This can still be attributed to the lack of cell proliferation in the last forming digits, as Alberch predicted.

Recent models for polydactyly formation in vertebrates lend further support to Alberch’s ideas on the generative constraints in digit formation. In this case, it is not the loss of digits but the addition of surplus digits that highlights the relationship between cell number and digit formation. Several different kinds of mutations affecting the Shh pathway, for instance, maintain cell proliferation in the area where the last digits form and lead to (predominantly) preaxial digit addition in cats [40]. Cellular automata models based on reaction–diffusion algorithms also show that reaction rates and cell number play a significant role in forming supernumerary digit-like regions of activation [41]. Notably, in the same way as digit loss is often an all-or-nothing event, digit addition also exhibits nonlinear behaviours.

Regardless of whether the underlying developmental mechanisms are fully understood, the power of Alberch’s model of digit loss in amphibians lies precisely in its testability. The model is formulated at a morphogenetic level [42]. It cannot be reduced to molecular parameters, and it can be contrasted with selectionist explanations of the same evolutionary transitions. In addition, an understanding of the cell and tissue behaviours of the developing limb permits it to be predictive about the evolutionary patterns that will occur when the system is affected by any perturbation that has a bearing on cell number. One of the main strengths of Alberch’s model resides in its capacity to inspire other models for the evolution of development. It allows the utilization of realistic generative parameters to construct the morphospace of the character under examination and to trace evolutionary patterns [43]. Following Alberch’s pioneering work, several developmental morphospaces have been proposed, encompassing various features such as the beaks of Darwin finches [44] or vertebrate limb and digit patterns [41]. One of the most fruitful endeavours has been the development of a computational model for the evolution of mammalian teeth, which is grounded in the genetic and cellular interactions underlying development [45].

Besides comparative morphology and the experimental probing of developmental potentialities, another one of Alberch’s sources of information for the concept of constraints was teratology ([46], this issue). Reviving an old tradition dating back to Paré and Saint Hilaire, he saw what he loved to call ‘natural monsters’ as ‘evidence for internal constraint in development and evolution’ [23]. Teratologies show that even major disturbances of embryonic development generate non-random outcomes revealing morphogenetic regularities. In his 1989 paper and other publications, Alberch discusses examples from aortic arches in rodents, homeotic transformations in *Drosophila*, cyclopia and head bifurcations in guinea pigs and humans, conjoined twins, as well as digital anomalies in vertebrate limbs, emphasizing the far-reaching trans-specific parallelisms in the genesis of such forms (for more recent examples, see [47]). It is important to note that by invoking these cases, Alberch did not mean to imply that such teratologies were in themselves variations with evolutionary potential in the sense of ‘hopeful monsters’. On the contrary, he argued that malformations represent an ideal study system for revealing developmental propensities, precisely because they arise in the absence of adaptive advantages and exhibit recurrent regularities despite the strong negative selection they must be subjected to.

**Figure 4.**
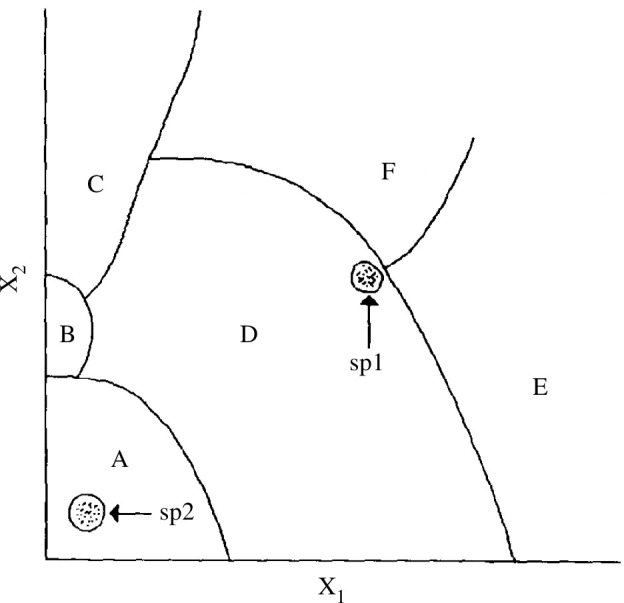
Illustration of a hypothetical parameter space composed of six phenotypes, A, B, C, D, E and F, determined by the developmental interactions of two parameters *x*_1_ and *x*_2_. Reproduced from Alberch [26], used with permission from Springer. Alberch uses this figure to illustrate four general conclusions about the properties of developmental systems (pp. 7–8). (i) Numerous combinations of parameter values can yield the same phenotype, indicating a lack of one-to-one correlation between changes in parameter values (whether genetic or environmental) and resulting phenotypic transformations. (ii) The stability of a given phenotype correlates with the extent of its area in parameter space. (iii) Lines represent sets of critical (*x*_1_*, x*_2_) values, serving as transformative boundaries among phenotypes. (iv) The stability of a specific population of phenotypes (species 1, sp1, and species 2, sp2, in the figure) hinges on their location within the parameter space.

Based on these observations, Alberch argued that the phenotypic variation that becomes available in populations—and that will be exposed to natural selection—is neither random nor uniform. It is not random because of the inherent tendencies of developmental systems to generate recurrent morphogenetic patterns in an organized manner, i.e. changes along certain variational parameters are more likely to occur than along others. And it is not uniform, because many parameters of developmental systems comprise boundaries of homeostatic regulation. When a system is pushed over these limits, a new—but not haphazard—morphogenetic configuration can arise abruptly. Teratologies confirm instances of discontinuous variation, as in cases of digit loss or digit gain, whereas others represent the outcome of continuous variation, such as the cases of head malformation. The reason for this is that most developmental processes are self-regulating and homeostatic, which Alberch captured in his theoretical concept of the parameter space (see figure 4). The fact that this interpretation of constraints included the possibility of phenotypic discontinuity and, thus, countered the gradualist prerequisite of standard evolutionary theory, is often overlooked.

Alberch’s internalist conception of organizing development was one of the initiating moments in the origin of mechanistic evo-devo: ‘I focus on the internal rules that control the appearance of morphological variation, on the mechanistic basis of such rules, and on the evolutionary consequences of this internally determined order’ [23, p. 28]. But at the theoretical level, this stance provoked controversial debate, and the notion of constraint underwent significant modifications over the following decades. Initial resistance from the population genetic faction was strong but abated somewhat with the adoption of the constraints concept by John Maynard-Smith and others in a landmark paper entitled ‘Developmental constraints and evolution’ [48]. The paper was based on a Mountain Lake Conference, and its coauthors, Russ Lande, Stuart Kauffman and Lewis Wolpert, among others, also included Pere Alberch. The paper established a widely adopted definition of developmental constraints as ‘biases on the production of variant phenotypes, or limitations on phenotypic variability, caused by the structure, character, composition or dynamics of the developmental system’ (p. 266). At the same time, the text illustrates the struggle among its authors between the internalist and the externalist position, as it constantly returns to the genetic grounding of evolution, despite the fact that the kinds of constraints spelled out in the definition are largely nongenetic. Quite paradoxically, the paper argued that developmental constraints ‘undoubtedly play a significant role in evolution’ (p. 265) but at the same time sowed doubt about their importance in relation to selection and recommended ‘extreme caution in claiming that such constraints are responsible for evolutionary trends’ (p. 282).

The notion of constraints on organismal designs also reanimated the discussion on *innovation* and *novelty* in evolution and on whether the material and dynamical properties of development could be decisive determinants in their origination. The idea that variation and innovation are causally distinct has been voiced many times in evolutionary history, but only after development was seriously reconsidered in evolutionary biology, phenotypic novelty became empirically accessible [10,49]. Alberch did not dwell extensively on this issue but was again instrumental in the characterization of a neglected evolutionary phenomenon. It emerged as a corollary of his parameter space concept and the notion that discontinuities are inherent to developmental variation [23]. In a similar vein, certain classes of novelties have been considered by-products or side effects of directional selection that may destabilize ontogenetic homeostasis and push generative systems towards the transgression of developmental boundaries [26,50,51]. Such threshold phenomena were observed by Alberch & Gale [38] in the comparative and experimental variation of amphibian digits and are corroborated by the frequent occurrences of polydactyly in cats [40]. Another example is the shift of inductive capacity of somatic and visceral layers of the lateral plate mesoderm contributing to the emergence and positioning of paired appendages in vertebrate evolution [52].

An even more basic aspect of constraints and novelty also explored by Alberch is the physics of development, or the relationship between ‘mechanics, morphogenesis and evolution’ [53]. The physical properties of cells and tissues, such as adhesivity, viscosity, elasticity, tensegrity or phase separation, represent essential mechanistic links between molecular phenomena and form generation, the effects of which can be traced back to the emergence of multicellularity and the origin of developmental systems [54]. In the early 1980s, Alberch collaborated with pioneers of the mechanical investigation of embryogenesis such as George Oster and Gerret Odell, in the creation of the first mechanical models of how cells and tissues form and change during development, including the invagination of embryonic tissues due to the contractile properties of the cellular cytoskeleton [55].

Furthermore, in advanced forms of development, physics has a central role in the origination of new characters. Mechanotransduction pathways, for instance, can affect gene expression and developmental reactions via the alteration of compressive force or tension. An example is the pharyngeal jaw apparatus in Cichlidae and Labridae, two families of fish that evolved a novel synovial joint between the upper pharyngeal jaws and the ventral surface of the neurocranium. The evolutionary decoupling of skeletal elements accompanied by changes in muscle vector orientations and pressure forces was shown to elicit the formation of the new joints in both species independently [56]. Molecular studies of mechanosensing in cichlid jaw formation have identified genes that act as mediators between bone formation, developmental plasticity and evolution and, at the same time, reveal the predictability of the evolutionary responses to environmental induction [57].

The results of developmental investigations of evolutionary events have led to the recognition that development is a key factor in the origin of morphological novelties [58,10]. As a consequence, the question arose whether adaptive evolution is able to bypass or needs to overcome and break developmental constraints [48,59] and whether a transition between adaptive peaks of fitness harbours the ultimate key to innovation [60]. The relationship between emergent development and adaptive variation in the explanation of innovation and novelty became a core topic in discussions of the role of development in evolution [61].

## 4. Constraints in current conceptual debates

In addition to the issue of evolutionary novelty, developmental constraints have become part of several metatheoretical discourse lines in the theory and philosophy of biology. One major topic concerns the question of whether constraints assume only a restricting, sometimes called ‘negative’ role in evolution, or whether they can also have ‘positive’ or enabling effects. This disjunctive distinction was somewhat artificially exaggerated because neither in Alberch’s understanding nor in Maynard-Smith *et al*.’s definition were constraints seen as mere limitations but rather as tendencies that determine morphological change. Constraint in the sense of generative bias was primarily meant as a productive concept, used to explain the causes for certain forms to appear rather than others, and limitation was only one aspect in this. It was to a large extent a rhetorical debate [62], but under the impression of the perceived negative connotation, and with new proposals of ‘facilitated variation’ arising [63], the term ‘developmental bias’ became more fashionable [64]. It more explicitly expressed the notion that development could have both restricting and enabling roles.

Broader notions like ‘variational properties’ avoid indirect references to selection [65,66]. Salazar-Ciudad and colleagues provide a classification of these mechanisms and argue that each type contributes to morphological evolutionary transitions [67]. Stuart Newman, for instance, has introduced the concept of ‘developmental patterning modules’ (DPMs) [68]. DPMs consist of gene products from the ‘developmental-genetic toolkit’ coupled with physical processes such as cohesion, viscoelasticity and spatiotemporal heterogeneity driven by activator–inhibitor interactions. More generally, these new efforts in unravelling and classifying general developmental mechanisms offer an alternative conceptual framework for explaining evolution. A large part of evo-devo research follows the tradition of comparative embryology, reinstantiated as comparative developmental genetics, aiming to comprehend how development has evolved. Conversely, another approach, sometimes referred to as ‘devo-evo’ [69–71], seeks to understand how development has influenced evolution. Alberch’s work navigated between these two approaches, exploring general morphogenetic principles that shape the evolution of animal form through case studies such as cell number changes and digit loss in limb buds.

Alberch’s idea that certain forms will appear in evolution, rather than not appearing, is also captured by the more recent concept of *inherency* (see [72] for an updated review). Its emphasis is more specifically on the physical forces and generic processes involved in organizing cells and tissue masses in development and that inevitably give rise to recurrent structural motifs such as cavities, spheres, rods, layered or branching structures. Phenotypic evolution is thought to result from elaborations of these generic themes, which may include their genetic routinization and fixation, but their causal origins would be based on the physical properties of developmental materials. Inherency defies the notion of a strict genetic programming underlying all of development and challenges the modern synthesis paradigm that all of evolution is based on repeated cycles of selection for improved fitness. Instead, the first origins of structures and the developmental kernels for later additions of certain classes of morphological novelties will depend on physical propensities and, in this sense, would be non-Darwinian. Alberch would probably not have gone this far, but the concept of inherency conforms with his notion of intrinsic tendencies in the production of variant phenotypes. Both ideas are in agreement with a theoretical distinction already made by Cope [73] in his renowned dictum ‘the law by which structures originate is one thing; those by which they are restricted, directed, or destroyed is another thing’.

In evolutionary theory, the new internalist framework had significant consequences for how the generation of variation in evolution was perceived. Whereas in the standard view, the focus in answering this question had been on natural selection and on whether sufficient genetic variation was available for the modification of a phenotypic character, now the focus was on the generative rules that determine the variation presented to natural selection. Alberch argued that ‘the interplay between genetic indeterminacy and resilient developmental rules suggests the likely recurrence of similar morphological variation and the generation of a biased subset of phenotypes upon which natural selection or population stochastic factors can operate’ [38, pp. 19–20]. In this view, developmental interactions do not constrain natural selection but how genetic mutations are expressed at the morphological level. This led the philosopher Ron Amundson [74] to distinguish between constraints on adaptation and constraints on form—which would be indifferent to adaptation and were only a consequence of morphogenetic potentialities. According to this view, generative constraints on form cannot be reduced to functional constraints.

Developmental constraint, facilitation, bias and inherency belong to the same class of explanatory concepts in evolutionary biology that allows philosophers to characterize evo-devo as a science of dispositions [75,76]. Other concepts related to this dispositionalist style of thinking include modularity, robustness, plasticity and evolvability. The evolutionary consequences are multifold. On the one hand, the developmentalist approach highlights the fact that the discrete nature of phenotypic evolution requires an explanation and needs to be reflected in evolutionary theory. The search for the rules that govern phenotypic transitions, and define a probability space of phenotypic outcomes, has become a legitimate task in evolutionary biology. Such rules can be identified by comparative, experimental, teratological and *in silico* analyses, and are amenable to formalization for theoretical integration. It also became clear that not all phenotypic variation needed to be minute, continuous and incremental. A developmental conceptualization of variation permits the occurrence of nonlinear transitions. Overall, the new approach made clear that a generative component was required in evolutionary theory. Phenotypic variation must not be treated merely as an extrapolation of genetic variation but requires an understanding of the interplay of internal organizing properties obeying generative rules with external influences of which natural selection is but one. In the maze of these conceptual adjustments, the very notion of selection undergoes a transformation. The most visible contribution of this revised approach to evolutionary theory may be seen in the work on evolvability.

## 5. From constraints to evolvability

While *evolvability*, like many topics in evolutionary biology, has roots tracing back to Darwin, it only materialized as a distinct research programme in the late 1990s, experiencing exponential growth since the mid-2000s. Research on evolvability has diversified since across various domains of biology, earning recognition as a key ‘expander’ of the evolutionary synthesis (see [77]). Hendrikse and colleagues confronted the increasing confusion of evo-devo with comparative developmental biology by arguing that its most valuable contribution lies in understanding how development causally shapes evolution. They proposed that evolvability should represent the ‘proper focus’ and the central question that defines the entire field [78]. Others have contested the narrowing of the conceptual richness of evo-devo to a single question and warn of the potential confusion of a developmental understanding of evolvability with the pervasive population genetic usage in terms of the (genetic) ability of a population to respond to natural selection [79]. Different interpretations of the concept of evolvability persist in different areas of evolutionary biology, but here we will proceed by examining Alberch’s contribution to its origination.

Alberch played a pivotal role in laying the conceptual groundwork for a developmental perspective on evolvability, sparking the interest of many biologists engaged in other domains of evolutionary biology, such as evolutionary systems biology. In the 1988 Dahlem Conference, he participated in a group discussion on the evolution of evolvability [80]. The discussion was coordinated by Steve Arnold and included participants as diverse as Pere, Richard Dawkins, John Maynard Smith, Elisabeth Vrba, Günter Wagner and David Wake. The group report touched upon nearly every topic later addressed in the literature on evolvability, ranging from the significance of developmental constraints to the various levels of selection. It amalgamated diverse perspectives on evolvability and its evolution, which were later elaborated upon by some of the group participants in distinct ways. In 1991, Alberch published in the journal *Genetica* a short review entitled ‘From genes to phenotype: dynamical systems and evolvability’, which constitutes the foundational text of a developmental perspective on evolvability and incorporated several concepts that subsequently appeared in key works of evolvability research.

Notably, Alberch introduced the *GP map* as the key conceptual tool for the study of evolvability [81]. The GP map was initially proposed by Waddington [82] and Lewontin [83] (as reviewed by Hallgrímsson *et al*.[84]) to refer to the structure of the relationship between genetic variation and phenotypic variation. Since early on in his career, Alberch had been dedicated to developing new mathematical models and visual representations for the ‘nonlinearities and thresholds in the mapping functions between genes and phenotypes’ [13]. In his 1991 paper, he recovers this theoretical approach, modelled with the formal tools of dynamical systems theory, to investigate how characteristics of developmental systems influence evolvability and its evolutionary trajectory (see figure 4). Since Alberch’s seminal paper, the notion of the GP map and its relation to evolvability have evolved into a cornerstone of evolutionary biology and its various branches, encompassing evolutionary quantitative genetics, computational molecular evolution and evo-devo.

In these different fields, evolvability is attributed to different bearers and is studied under different selection regimes and time scales [85]. Alberch’s contribution shaped the articulation of developmental approaches to evolvability. In his 1991 paper, he attributes the capacity for evolution not to populations, but instead defines an ‘evolvability potential’ determined ‘by the overall properties of the dynamical system’ (p. 9). Thus, evolvability emerges as a characteristic of developmental systems that enhances their capacity for evolutionary change. Moreover, Alberch recognizes that within this definition, evolvability itself can evolve, implying ‘a new level of selection, one that does not act on the phenotype nor on the genotype, but rather on the emergent properties of developmental systems’.

Over the subsequent decades, research on developmental evolvability was concerned with identifying the evolutionarily significant global properties outlined by Alberch. In the 1991 paper, he emphasized the interplay between stability and variability, suggesting that pattern-generating systems displaying the right balance between the two were favoured by selection. Wagner and Altenberg’s seminal paper in 1996 linked the study of evolvability with the GP map, proposing that the modularization of genetic effects on traits promotes evolvability. Subsequently, Kirschner & Gerhart [86] recovered Alberch’s original approach and identified five properties—versatile protein elements, weak linkage, compartmentalization, redundancy and exploratory behaviour—that confer robustness and flexibility on developmental processes, thereby facilitating morphological evolution. In the early twenty-first century, the examination of the interplay between stability and variability emerged as a central theme in investigations of the connection between robustness and evolvability [87].

Alberch’s reflection on evolvability highlights crucial aspects of his thought on evo-devo. First, the developmental approach to evolvability underscores the positive role of developmental constraints discussed above. The inclusion of selection into the definition of evolvability transformed the concept into one more amenable to acceptance by the mainstream evolutionary community, despite the initial resistance to linking constraints with limitations on selection [62,77]. Second, the properties of GP maps affecting evolvability through their interplay with general selection regimes, such as the balance between robustness and variability of developmental systems envisaged by Alberch, have a wider degree of generality than developmental rules that apply to specific lineages. This approach invalidates the interpretation of evo-devo as a discipline that can only address local, proximate causes of evolution. But today, most works endorsing a developmental approach to evolvability tend to focus on the mapping of gene or protein expression onto phenotypic variation, or on the structural properties of gene regulatory networks. This re-emphasis of the molecular level has raised scepticism among organism-oriented biologists (e.g. [58]), who, like Alberch, attribute evolvability to the emergent properties of developmental systems. Explorations of the relevant roles of these properties in evolvability have been limited, but recent promising examples are moving in this direction (see [88]).

Finally, Alberch’s developmental perspective on evolvability demonstrates how internalist approaches do not necessarily entail a disregard for the adaptive dimension of evolution. Rather than denying selection, it involved an assessment of its relative significance and a reconceptualization of the role of selection itself in the evolution of development. In his early work, Alberch had downplayed selection as ‘basically stabilizing, being responsible for “pruning out” the nonfunctional morphologies and for determining the differential survival of morphological types’ [13, p. 319]. But his later reflections on the properties that make developmental systems selectable turn to a more elaborate idea of selection in which the notion of ‘biological function’ transcends the role of morphological characters as mere subjects of adaptation via natural selection. On the one hand, he argued that functional constraints result from the interplay among the constituent parts of the organism [89]. This idea connects to the notion of internal selection originally advanced by Whyte [90] and later elaborated by Riedl [91] in his concept of ‘burden’, which addressed the functional interdependencies among traits. Internal selection concerns the selection of traits that can be integrated early in development without compromising the functional integrity of embryos. Just like the ability to vary, developmental viability is a condition that operates under a wide range of selectional regimes [92]. On the other hand, the developmental view of evolvability and its evolution allowed Alberch to interpret developmental systems as the true locus where selectable morphological variation is generated. Whereas Alberch’s consideration of functional factors remains underexplored in evo-devo, it has gained increasing significance since the turn of the twentieth century [93].

## 6. Conclusion

While Alberch’s work was a major contribution to the formation of evolutionary developmental biology as a discipline, his approach was eventually superseded by the empirical achievements of the molecular revolution in evolutionary biology. But his theoretical elucidation of fundamental concepts in evo-devo research, including the interaction between developmental constraints and natural selection, the recognition of the morphogenetic level as the appropriate locus of developmental causation, the emergence of evolutionary novelties and the concept of evolvability, has continued to exert a lasting influence that has kept his ideas alive after his untimely death in 1998. Alberch’s work has had an impact beyond evo-devo, from the evolution of cognition [94] and the evolution of culture [95], to new conceptualizations of the evolutionary framework [79]. Just as the molecular revolution in the 1990s enabled a thorough exploration of the genetic connections and disconnections that underlie living diversity, the more comprehensive integration envisioned by Pere Alberch of experimental, mathematical and theoretical approaches is still paving the way for integrated, multiscale analyses of development that elucidate the evolution of this diversity.

## Data Availability

This article has no additional data.
